# Microwave-Assisted Synthesis of Trazodone and Its Derivatives as New 5-HT_1A_ Ligands: Binding and Docking Studies

**DOI:** 10.3390/molecules24081609

**Published:** 2019-04-23

**Authors:** Jolanta Jaśkowska, Przemysław Zaręba, Paweł Śliwa, Edyta Pindelska, Grzegorz Satała, Zbigniew Majka

**Affiliations:** 1 Institute of Organic Chemistry and Technology, Faculty of Chemical and Engineering and Technology, Cracow University of Technology, 24 Warszawska Street, 31-155 Cracow, Poland; pzareba@chemia.pk.edu.pl (P.Z.); psliwa@chemia.pk.edu.pl (P.Ś.); 2 Department of Analytical Chemistry and Biomaterials, Faculty of Pharmacy with the Laboratory Medicine Division, Medical University of Warsaw, Banacha 1, 02-093 Warsaw, Poland; edyta.pindelska@wum.edu.pl; 3 Department of Medicinal Chemistry, Institute of Pharmacology-Polish Academy of Sciences, 12 Smętna Street, 31-343 Cracow, Poland; satala@if-pan.krakow.pl; 4 TM Labs Sp. z o.o., Al. W. Beliny-Prażmowskiego 14, 31-514 Kraków, Poland; zbigniew.majka@tmlabs.pl

**Keywords:** trazodone derivatives, trazodone synthesis, microwave (MW)-assisted synthesis, serotonin receptor ligands, 5-HT_1A_ ligands, long-chain arylpiperazines

## Abstract

Trazodone, a well-known antidepressant drug widely used throughout the world, works as a 5-hydroxytryptamine (5-HT_2_) and α_1_-adrenergic receptor antagonist and a serotonin reuptake inhibitor. Our research aimed to develop a new method for the synthesis of trazodone and its derivatives. In the known methods of the synthesis of trazodone and its derivatives, organic and toxic solvents are used, and the synthesis time varies from several to several dozen hours. Our research shows that trazodone and its derivatives can be successfully obtained in the presence of potassium carbonate as a reaction medium in the microwave field in a few minutes. As a result of the research work, 17 derivatives of trazodone were obtained, including compounds that exhibit the characteristics of 5-HT_1A_ receptor ligands. Molecular modeling studies were performed to understand the differences in the activity toward 5-HT_1A_ and 5-HT_2A_ receptors between ligand **10a** (2-(6-(4-(3-chlorophenyl)piperazin-1-yl)hexyl)-[1,2,4]triazolo[4,3-a]pyridin-3(2*H*)-one) (5-HT_1A_ *K*_i_ = 16 nM) and trazodone. The docking results indicate the lack of the binding of ligand **10a** to 5-HT_2A_R, which is consistent with the in vitro studies. On the other hand, the docking results for the 5-HT_1A_ receptor indicate two possible binding modes. Crystallographic studies support the hypothesis of an extended conformation.

## 1. Introduction

**Figure 1:**
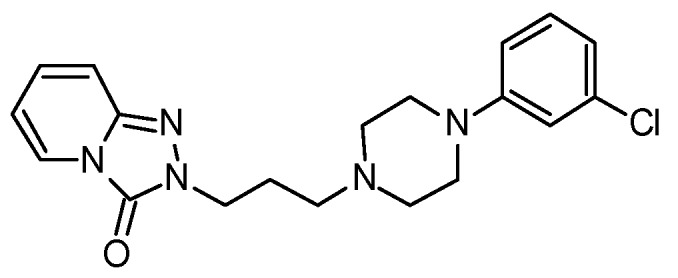
Chemical structure of trazodone (2-{3-[4-(3-chlorophenyl)piperazin-1-yl]propyl}[1,2,4]triazolo[4,3-a]pyridin-3(2*H*)-one).

Trazodone (Figure 1) is a well-known antidepressant drug used since the 1960s, and it is widely applied throughout the world. The pharmacological effect of trazodone is due to serotonin reuptake inhibition and 5-hydroxytryptamine (5-HT_2_) receptor antagonism, whose activation usually leads to insomnia, anxiety, psychomotor agitation, and sexual dysfunction.

In the most commonly described methods of obtaining trazodone, the reaction is carried out using 2-(3-halopropyl)[1,2,4]triazolo[4,3-a]pyridin-3(2*H*)-one and 1-(3-chlorophenyl) piperazine hydrochloride, e.g., in toluene in the presence of triethylamine for about 3 h [1,2] or in acetonitrile in the presence of potassium carbonate for 24 h [3]. There are also known processes for the preparation of trazodone in the reaction of 1-(3-bromopropyl)-4-(3-chlorophenyl)-piperazine and [1,2,4]triazolo[4,3-a]pyridin-3(2*H*)-one. The reaction is carried out in two steps in dioxane, where the total reaction time is about 21 h [4], or in isopropyl alcohol, in the presence of TBAB (tetrabutylammonium bromide) and sodium carbonate [5]. Other known methods involve the use of ester, i.e., 3-(3-oxo-[1,2,4]triazolo[4,3-a]pyridine-2(3H-yl)propyl methanesulfonate as a substrate in the reaction with 1-(3-chlorophenyl)piperazine, to obtain the expected product within 16 h of heating in acetonitrile in the presence of potassium carbonate [3].

In summary, the methods of the synthesis of trazodone, which are known so far, require solvents such as acetonitrile, toluene, dioxane, or isopropyl alcohol, and the synthesis time of the product varies from several to several dozen hours.

As already mentioned, trazodone is a potent serotonin 5-HT_2A_ and α_1_-adrenergic receptor antagonist.

Although trazodone is a well-known antidepressant, its pharmacological activity is not fully understood, and it is thought to have more than one mechanism of action. There is ample evidence that suggests that the antidepressant activity of trazodone involves serotonin type 2 (5-HT_2_) receptor antagonism and the inhibition of serotonin transporter, which results in agonistic effects against 5-HT_1A_. As a result, 5-HT_1A_, 5-HT_2_, and other receptors (5-HT_6_, 5-HT_7_, and D_2_) play an important role in these diseases of the central nervous system [6,7,8,9,10,11,12,13,14,15]. In addition, 5-HT_1A_ receptor ligands are of great interest due to the fact that new therapeutic targets were identified, i.e., prostate cancer treatment, and gastrointestinal and cardiopulmonary disorders [16].

We recently reported the synthesis of a number of hexylarylpiperazine derivatives, which showed high affinity for 5-HT_1A_ receptor [17,18]. As a continuation of this study and in order to test whether the extension of the trazodone linker will change the activity profile, we present here the design, synthesis, and biological evaluation of new derivatives of trazodone as potential 5-HT_1A_ ligands. In the research work, we also attempted to adapt our method of synthesis under microwave radiation [5,19], in the synthesis of trazodone itself, as well as of its substrates and its derivatives.

## 2. Results and Discussion

### 2.1. Synthesis of Trazodone

**Scheme 1:**
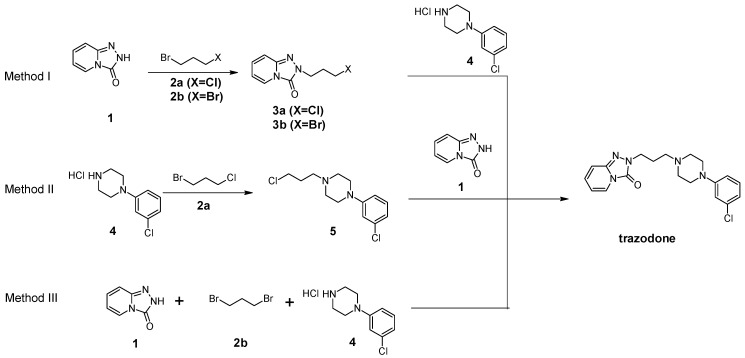
Methods for obtaining trazodone.

Our research shows that trazodone can be successfully obtained in the reaction of 2-(3-halopropyl)[1,2,4]triazolo[4,3-a]pyridin-3(2*H*)-one (**3a/b**) and 1-(3-chlorophenyl) piperazine hydrochloride (**4**), carried out in the presence of potassium carbonate as a reaction medium, a PTC (phase transfer catalyst) in the microwave radiation field (Scheme 1, Method I). The same process conditions can be used successfully to synthesize trazodone with the reaction of 1,2,4-triazolo[4,3-a]pyridin-3(2*H*)-one (**1**) with chloropropyl-2-chloroarylpiperazine (**5**) (Scheme 1, Method II). In addition, these conditions can be also used in a “one-pot” reaction, without isolating intermediates (Scheme 1, Method III).

**Scheme 2:**
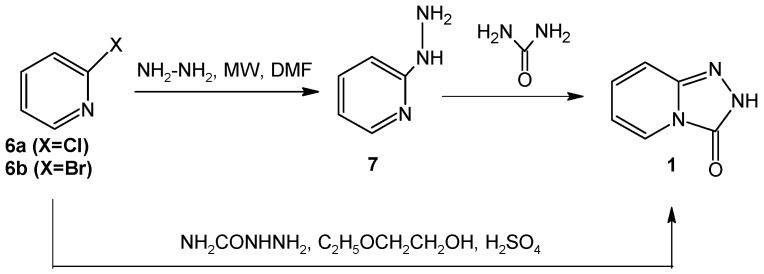
Methods for obtaining 1,2,4-triazolo[4,3-a]pyridin-3(2*H*)-one (**4**) under microwave (MW) radiation.

The research started with the synthesis of 1,2,4-triazolo[4,3-a]pyridin-3(2*H*)-one (**1**) using two methods (Scheme 2). The first one concerns a two-step process involving the reaction of 2-halopyridine (**6a/b**) with hydrazine in the first stage (yield 14%) in dimethylformamide (DMF) for 120 s. Bearing in mind the low efficiency, the reaction was also carried out under conventional conditions by heating the substrates in ethanol for 25 h [3], which resulted in a 50% yield. In the case of the synthesis of **7** from **6a**/**b**, the higher yield was obtained by classical synthesis. In the next step, the obtained compound **7** was reacted with equimolar amount of urea (yield 49%) or with molar excess (yield 75%) in a solvent-free condition for 50 s. The obtained results, especially in the second stage, are very interesting because the analogous reaction under conventional conditions described in the literature takes 2 h and allows the synthesis of compound **1** with a lower yield (57%) [20].

The second method for the synthesis of **1** in a one-step reaction involves the use of semicarbazide in 2-ethoxyethanol (yield 43–48%). All reactions were carried out under microwave radiation, which is a new way of obtaining 1,2,4-triazolo[4,3-a] pyridin-3(2*H*)-one (**1**) and which is yet to be described in the literature.

#### 2.1.1. Method I

**Scheme 3:**
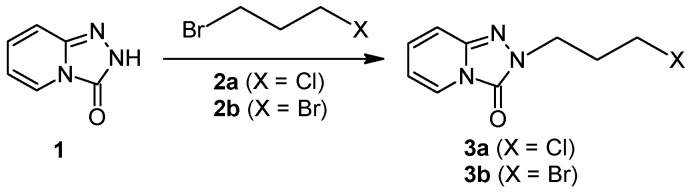
Synthesis 2-(3-halopropyl) [1,2,4] triazolo [4,3-a] pyridin-3 (2*H*) -one (**3a/1b**) under MW radiation.

The synthesis of trazodone was started using the method I, which was based on *N*-alkylation of the previously obtained 1,2,4-triazolo[4,3-a]pyridin-3(2*H*)-one (**1**) with dihalogenopropane (**2a/b**) (Scheme 3).

The reactions were carried out under microwave radiation in the presence of K_2_CO_3_ and PTC catalysts (TBAB). Table 1 contains the results of the research including the impact of the solvent (acetonitrile (ACN) and DMF) on the yield and time of obtaining **3a/b**.

The studies showed that, when the reaction is carried out with DMF or ACN under the same conditions, the obtained efficiency is almost four times better with ACN (Table 1, entries 1 and 2); therefore, further experiments were carried out with this solvent only. Surprisingly, a subsequent study showed that, when the amount of the solvent was decreased, or even completely eliminated, this did not cause significant differences in the performance (Table 1, entries 2, 3, 4, 7, and 8). The replacement of halogen in the substrate **2a** on **2b** caused a slight 17% drop in yield (Table 1, entries 4 and 7). In the case of the reaction carried out in a Magnum II reactor in a closed polytetrafluoroethylene (PTFE) tube, similar efficiency was obtained, but it was necessary to extend the time of the process (Table 1, entries 4 and 5).

**Scheme 4:**
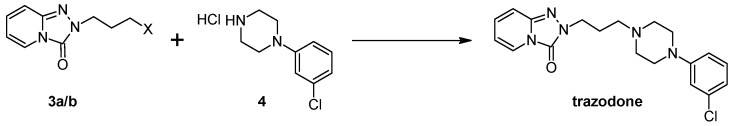
Synthesis of trazodone with method I under MW radiation.

In the next stage of the synthesis of trazodone with method I, the obtained 2-(3-halopropyl)[1,2,4]triazolo[4,3-a]pyridin-3(2*H*)-one (**3a/b**) reacted with 1-(3-chlorophenyl)piperazine hydrochloride (**4**) (Scheme 4).

The synthesis of trazodone with the method I was carried out with potassium carbonate and PTC (TBAB—tetrabutylammonium bromide, TEAC—tetraethylammonium chloride, and DABCO—1,4-diazabicyclo[2.2.2]octane). The reactions were carried out under microwave radiation. During the experiment, we observed that the addition of a small amount (<10% weight) of DMF or ACN was beneficial for the duration of the process, but the reaction also took place under completely solvent-free conditions (Table 2, entries 5, 6, and 11). The obtained results for the synthesis of trazodone with method I showed that the reaction occurred in less than 5 min, while, in the presence of DMF or ACN, it occurred within 1–2 min. The highest yield was observed in the reaction with 2-(3-bromopropyl)-1,2,4-triazolo[4,3-a]pyridin-3-(2*H*)-one (**3b**) using 10% by weight DMF and TBAB as a PTC catalyst, which allowed obtaining a product with 98% efficiency in 1 min (Table 2, entry 2). When the TBAB was exchanged for TEAC or DABCO under the same conditions, the reaction time was longer by 2 min (Table 2, entries 3 and 4). The exchange of DMF for ACN caused a 13% decrease in yield (Table 2, entries 1 and 7).

In the case of the use of 2-(3-chloropropyl)-1,2,4-triazolo[4,3-a]pyridin-3-(2*H*)-one (**3a**), the amount of ACN in the range of 10–40% did not significantly affect the efficiency (Table 2, entries 8–10). For these reactions, higher yields were observed when ACN was used rather than DMF or H_2_O. The effect of pressure on the course of the reaction and the product yield were also evaluated by conducting the process in a closed PTFE tube in a Magnum II reactor. For pressures of 1 and 5 bar, similar results were obtained, while the increase of the pressure to 10 bar resulted in a decreased product efficiency by about 20% (Table 2, entries 14–16).

#### 2.1.2. Method II

**Scheme 5:**
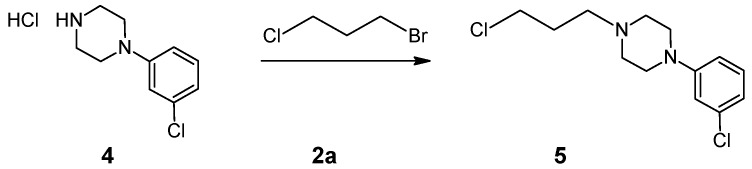
Synthesis 1-(3-chloropropyl)-4-(3-chloropheyl)piperazine (**5**) under MW radiation.

In the next stage of our research related to the synthesized trazodone with method II, 1-(3-chlorophenyl)-4-(3-chloropropyl)piperazine (**5**) was obtained in the reaction of 3-chloroarylpiperazine (**4**) with 1-bromo-3-chloropropane (**2a**) (Scheme 5) under microwave radiation.

The best results in the synthesis of 1-(3-chloropropyl)-4-(3-chlorophenyl)piperazine (**5**) were obtained by reacting with ACN in a Samsung microwave reactor, where the product was obtained with a yield of 88% within 40 s. (Table 3) This is a much better result than the analogous method described in the literature so far under conventional conditions where 1-(3-chloropropyl)-4-(3-chlorophenyl) piperazine (**5**) can be obtained with a 60% yield within 7 h [21].

**Scheme 6:**
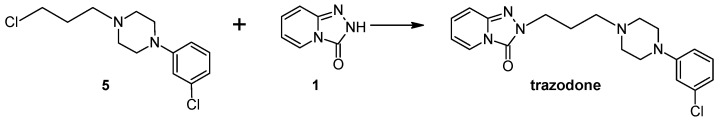
Synthesis of trazodone with method II under MW radiation.

To obtain trazodone with method II, the previously prepared 1-(3-chloropropyl)-4- (3-chlorophenyl)piperazine (**5**) was reacted with 1,2,4-triazolo[4,3-a]pyridin-3(2*H*)-one (**1**) (Scheme 6). The synthesis was carried out under microwave radiation in the presence of potassium carbonate, TBAB, and ACN.

The highest efficiency (92%) in the synthesis of trazodone with method II was obtained in the reaction for 80 s using about 15 wt.% ACN (Table 4, entry 4).

#### 2.1.3. Method III—One-Pot Synthesis

Trazodone was also obtained in a “one-pot” variant. The process was carried under microwave radiation by firstly heating 1,2,4-triazolo[4,3-a]pyridin-3(2*H*)-one (**1**) with 1-bromo-3-chloropropane (**2a**) for 50 s, followed by the addition of 1-(3-chlorophenyl)piperazine (**4**) for another 90 s. The expected product was obtained with a yield of 31%. The synthesis was also performed in the CEM Discover SP reactor (100 W), carrying out the process for 30 s in the first stage and for 60 s in the second, which allowed obtaining trazodone with a yield of 71%.

We also tested the variant in which all of the reactants, 1,2,4-triazolo [4,3-a]pyridin-3(2*H*)-one (**1**), 1-bromo-3-chloropropane (**2a**), and 1-(3-chlorophenyl)piperazine (**4**), were treated with microwave radiation, but in this case trazodone was not observed in the post-reaction mixture.

To summarize the synthesis of trazodone under microwave radiation, it can certainly be assumed that this method is applicable at every stage of the synthesis. This method, in comparison to conventional methods, increases the yield of the obtained products, shortens the time of the synthesis, excludes or reduces the amount of solvents used in the process, and reduces the amount of energy.

Bearing in mind that the highest yields of trazodone were obtained with method I, as well as the fact that this process can be quite easily scaled-up, we decided to synthesize trazodone derivatives according to this method.

### 2.2. Synthesis of Trazodone Derivatives

**Scheme 7:**
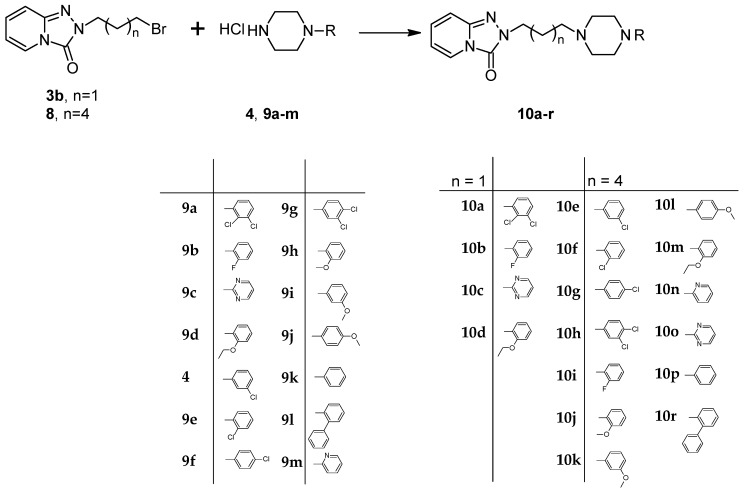
Synthesis of trazodone derivatives with method I.

The synthesis of trazodone with method I also works very well in the synthesis of its derivatives (Scheme 7, Table 5) (**10a–r**). The reactions were carried out under similar conditions as the previous ones, i.e., potassium carbonate, TBAB, and a small amount of ACN. All of the syntheses were carried out in the SAMSUNG device (Table 6). Additionally, for the selected ligands, the synthesis in the CEM Discover SP reactor was also performed, giving slightly higher yields (**10e**, yield = 58%; **10o**, yield = 61%; **10r** yield = 66%). The obtained products were converted into hydrochlorides.

The derivatives of trazodone (**10a–k**) with a modified aryl substituent were obtained by the condensation reaction of 2-(3-chloropropyl)-1,2,4-triazolo[4,3-a]pyridin-3-(2*H*)-one (**4a**) with appropriate arylpiperazines **7**. As already mentioned in the introduction, and bearing in mind the high activity with 5-HT_1A_ receptors in ligands with the hexyl linker that we previously synthesized [17,18,19,22], hexyl derivatives of trazodone were also obtained (**10e–k**) (Scheme 7).

The progress of the reaction was monitored with thin-layer chromatography (TLC) and the purity of the resulting ligands was assessed with UPLC-MS. The structures were confirmed based on the analysis of data obtained from ^1^H NMR, ^13^C NMR, and infrared (IR).

### 2.3. Biological Evaluation of Trazodone Derivatives

The ligands obtained in the synthesis (**10a–r**) were tested in vitro for binding to D_2_, 5-HT_1A_, 5-HT_2A_, 5-HT_6_, and 5-HT_7_ receptors on the basis of the screening protocol described previously [17] (Table 6).

The in vitro results collected in Table 6 show that replacement of the 3-chloroarylpiperazine system in trazodone, as well as chain elongation, results in a decreased activity relative to the 5-HT_2A_ receptor. Interestingly, extending the chain by three carbon atoms, while maintaining the 3-chloroarylpiperazine system, resulted in a change in the pharmacological profile and binding to the 5-HT_1A_ receptor (**10e**). The majority of the synthesized hexyl ligands (Table 6, entries 5–18) provided a high affinity and selectivity for the 5-HT_1A_ receptor.

Interestingly, adding chlorine to this structure, at position 3 to position 4 (**10h**) resulted in a fourfold increase of the activity toward 5-HT_2A_ and 5-HT_6_. When there was only a substituent in the 4-position (**10g**), the activity was similar to that in the case of **10e** (Table 6). Among ligands with chlorine substitution, the compound with the substitution at position 2 (**10f**) had the least interesting activity profile. However, when chlorine was replaced with fluorine at this position, the binding and selectivity to 5-HT_1A_ increased significantly (**10i**). Among ligands with a hexyl chain and the arylmethoxy group linked to the piperazine moiety, ligands with substitution at positions 2 (**10j**) and 3 (**10**) had a much higher binding to 5-HT_1A_ compared to their 4-substituted analog (**10l**). Interestingly, if the methoxy group was replaced by an ethoxy group in position 2 (**10m**), selectivity increased significantly. Our research showed that the number of nitrogen atoms in the arylpiperazine system also strongly affected binding to the 5-HT receptors. When the ligand had two nitrogen atoms at positions 2 and 6, the activity was >100 nM for all receptors (**10o**). When there was only one nitrogen atom in the arylpiperazine system (**10n**), the activity with the 5-HT_1A_ and D_2_ receptors increased significantly, after which, when the nitrogen atoms were completely eliminated (**10p**), the compound was active only with 5-HT_1A_. Of all the trazodone derivatives obtained, the characteristics of 5-HT_1A_ receptor ligands were shown by **10e**, **10g**, **10h**, **10i**, **10m**, and **10p**. It is also worth noting that very interesting features as a dual 5-HT_1A_/_7_ ligand were shown by **10r**.

### 2.4. Molecular Modeling

**Figure 2:**
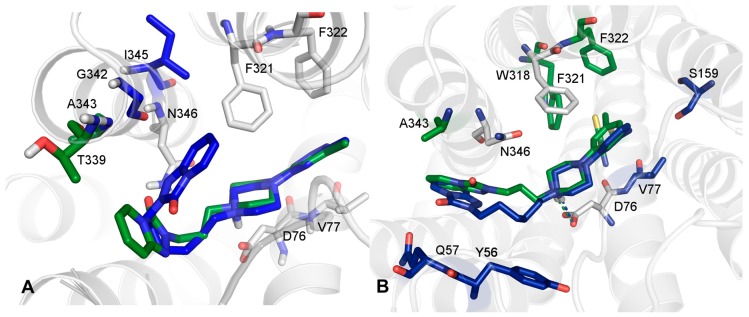
Two considered binding modes of **trazodone** (blue) and **10e** (green) in the homology model of serotonin receptor 5-hydroxytryptamine (5-HT_1A_). Only key residues of the receptor are marked as sticks, where those employed in the binding of both are uncolored and those specific in the ligands are colored.

In order to explain the effect of the alkyl linker elongation in trazodone on the affinity to receptors 5-HT_1A_ and 5-HT_2A_, we performed the molecular docking of the compound and representative ligand **10e** (entry 6 in Table 6). The results showed that **10e**, which contained the hexyl chain, did not bind to any used homology models of the serotonin receptor type 2A. In the case of the 5-HT_1A_R, two alternative binding modes were considered (Figure 2). For both, the key salt bridge with D76 and protonated piperazine moiety was observed. In the case of bent conformations, on the left of Figure 2 the chlorophenyl ring of both compounds formed a CH–π interaction with F321/F322 and the 1,2,4-triazolo[4,3-a]pyridin-3(2*H*)-one group interacted with N346. The main differences occurred in that last region. The elongated structure of **10e** caused contacts with G342 and I345, while the trazodone interacted with T339 and A343. The second arrangement in consideration, which was more coherent, was additionally supported by the results of crystallographic studies—the crystal structure of trazodone hydrochloride, CSD-CPTAZP [25] (B on Figure 2). In this case, the triazolopyridine ring expanded into the cavity between transmembrane domains (TMs) 2 and 7. This binding mode meant that only trazodone could interact with phenylalanine F322 and W318, which was the main reason for its greater affinity in comparison to a hexyl derivative. In this case, the ligand with the hexyl chain additionally had contact with residues from ECL2 (Q57 and Y56) and from TM5 (S159).

## 3. Experimental

### 3.1. Materials and Methods

The reactions in the microwave radiation field were mainly carried out in an Erlenmeyer flask in the Samsung M182DN device (300 W) and comparably in a closed PTFE tube in a Magnum II reactor (600 W) and CEM Discover SP reactor (100 W). All reagents from Sigma Aldrich (Poznan, Poland) and all organic solvents from POCH were of reagent grade and were used without purification. The progress of all reactions and purity of the synthesized compounds was confirmed by TLC, performed on Merck silica gel 60 F254 aluminum sheets (Merck, Darmstadt, Germany). Spots were detected by their absorption under ultraviolet (UV) light (λ = 254 nm). HPLC chromatograms were determined on a Perkin Elmer Series 200 HPLC with an XTerra RP C-18 (3.5 µm seed size, 4.6 × 150 mm) column and MeOH:H_2_O 1:1 eluent acidified with 0.1% formic acid as a phase (flow rate of 1 mL∙min^−1^) was used. IR spectra were taken on an FTS-165 spectrometer (FTIR Biorad). Melting points were determined on a Boetius apparatus and are uncorrected. The purification by HPLC was assessed by comparing product integration to overall integrated spectrum.

^1^H-NMR and ^13^C-NMR spectra were recorded at 300 MHz (Bruker Avance, Cracow, Poland) using tetramethylsilane (TMS; 0.00 ppm) and chloroform-d1; *J* values are in Hertz (Hz), and splitting patterns are designated as follows: s (singlet), d (doublet), t (triplet), m (multiplet).

The three-dimensional structures of the ligands were fully optimized at CAM-B3LYP/6-31G*19 level with the polarizable continuum model (PCM) (solvent = water) using Gaussian 09 software (Gaussian, Inc., Wallingford, CT, USA). The appropriate ionization states at pH = 7.4 ± 1.0 were assigned using MarvinSketch 18.29 (ChemAxon Europe, Budapest, Hungary). The AutoDock Tools was used to assign the bond orders, appropriate amino acid ionization states, and to check for steric clashes. The receptor grid was generated by centering the grid box with a size of 12 Å on D76 side chain. Automated flexible docking was performed using AutoDock Vina 1.5.6 [26]. The figures were prepared using PYMOL.

The homology models of the selected serotonin receptors, namely 5-HT_1A_ and 5-HT_2A_, were built on the D_3_ template (Protein Data Bank (PDB) identifier (ID): 3PBL), using a procedure described previously [27].

### 3.2. General Procedure for the Preparation of 2-Hydrazinopyridine (***7***) under Microwave Conditions (Samsung M182DN; 300 W)

Firstly, 2.50 g (0.016 mol, 1.5 cm^3^) of 2-bromopyridine (**6b**), 6.09 g (0.19 mol, 6 cm^3^) of hydrazine anhydrous (98%), and 10 cm^3^ of DMF were placed in a conical flask. Reactions were carried out under microwave radiation. The progress of the reaction was monitored by TLC (chloroform–methanol 9:1). After 2 min, DMF was evaporated in vacuo and 15 cm^3^ of water was added. The mixture was extracted 2 × 20 cm^3^ with methylene chloride. The organic extracts were combined and removed in vacuo to dryness; R_f_ = 0.41, yield (Y) = 14%.

### 3.3. General Procedure for the Preparation of 1,2,4-Triazolo[4,3-a]pyridin-3(2H)-one (***1***) 

#### 3.3.1. Preparation of 1,2,4-Triazolo[4,3-a]pyridin-3(2*H*)-one (**1**) from 7 under Microwave Conditions (Samsung M182DN; 300 W) 

Firstly, 500 mg (4.58 mmol) g of 2-hydrazinopyridine (**7**) and 275 mg (4.58 mmol) or 550 mg (9.16 mmol) of urea were placed in a conical flask. Reactions were carried out under microwave radiation. The progress of the reaction was monitored by TLC (chloroform–methanol 9:1). After 50 s, 15 cm^3^ of water was added to the mixture, after which the resulting product was filtered off on a Büchner funnel. The product was obtained with efficiency 49% in an equimolar reaction and 75% by reaction with a twofold molar excess of urea; R_f_ = 0.62, HPLC 97.9% (reaction with molar excess), t_M_ 1.95 min.

#### 3.3.2. Preparation of 1,2,4-Triazolo [4,3-a] pyridin-3(2*H*)-one (**1**) from **6a/b** and Semicarbazide under Microwave Conditions (Samsung M182DN; 300 W)

Firstly, 5.00 g (44 mmol) of 2-chloropyridine (**6a**), 9.81 g (88 mmol) of semicarbazide hydrochloride, 15 cm^3^ of 2-ethoxyethanol, and 0.1 cm^3^ of concentrated sulfuric acid were placed in a conical flask. Reactions were carried out under microwave radiation. The progress of the reaction was monitored by TLC (eluent chloroform–methanol 9:1). After 2 min, the reaction mixture was cooled to about 60 °C and 15 cm^3^ of water was added. The precipitate was filtered on a Büchner funnel and washed with about 50 cm^3^ of water. Y = 48%.

#### 3.3.3. Preparation of 1,2,4-Triazolo[4,3-a]pyridin-3(2*H*)-one (**1**) from 6b and Semicarbazide under Microwave Conditions (Magnum II Reactor; 600 W) 

Firstly, 350 mg (2.2 mmol) of 2-bromopyridine (**6b**), 491 mg (4.4 mmol) of semicarbazide hydrochloride, 0.75 cm^3^ of 2-ethoxyethanol, and 0.01 cm^3^ of concentrated sulfuric acid were placed in a PTFE vessel. Reactions were carried out under microwave radiation. The progress of the reaction was monitored by TLC (eluent chloroform–methanol 9:1). After 2 min, 0.75 cm^3^ of water was added to the reaction mixture. The precipitate was filtered on a Büchner funnel and washed with about 5 cm^3^ of water.

### 3.4. General Procedures for the Preparation of 2-(3-Halopropyl)[1,2,4]triazolo[4,3-a]pyridin-3 (2H)-one (**3a/b**)

#### 3.4.1. Synthesis 2-(3-Halopropyl)[1,2,4]triazolo[4,3-a]pyridin-3(2*H*)-one (**3a/b**) (Samsung M182DN; 300 W)

Firstly, 1.35 g (0.01 mol) of 1,2,4-triazolo[4,3-a]pyridin-3-(2*H*)-one (**1**), 2.50 cm^3^ (26 mmol, 4.09 g) of 1-bromo-3-chloropropane (**2a**)/2.65 cm^3^ (0.026 mol, 5.25 g) of 1,3-dibromopropane (**2b**), 320 mg (0.001 mol) of TBAB, 4.14 g (0.03 mol) of K_2_CO_3_, and the appropriate amount of acetonitrile (6, 3, 0.75, or 0.4 cm^3^)/DMF (5 cm^3^) were placed in a conical flask, after which the mixture was subjected to microwave radiation. The progress of the reaction was monitored by TLC (chloroform–methanol 9:1). The reaction times are summarized in Table 1. After the reaction, about 50 cm^3^ of water was added and the resulting product was filtered off on a Büchner funnel. The reaction yields are summarized in Table 1. The purity of the product obtained with the highest efficiency (Y = 92%) was confirmed using HPLC 99.3% t_M_ = 3.43 min.

#### 3.4.2. Synthesis 2-(3-Chloropropyl)[1,2,4]triazolo[4,3-a]pyridin-3(2*H*)-one (**3a**) (Magnum II Reactor; 600 W)

Firstly, 135 mg (1 mmol) of 1,2,4-triazolo [4,3-a] pyridin-3-(2*H*)-one (**1**), 0.25 cm^3^ (2.6 mol) of 1-bromo-3-chloropropane (**2a**), 32 mg (0.1 mmol) of TBAB, and 414 mg (3 mmol) of K_2_CO_3_ 0.075 cm^3^ of acetonitrile were placed in a PTFE vessel, after which the mixture was subjected to microwave radiation. The progress of the reaction was monitored by TLC (eluent chloroform–methanol 9:1). After 2 min, 5 cm^3^ of water was added and the resulting product was filtered off on a Büchner funnel. Y = 81%.

### 3.5. General Procedures for the Preparation of Trazodone According to Method I

#### 3.5.1. Preparation of Trazodone according to Method I under Microwave Conditions (Samsung M182DN; 300 W)

Firstly, 2.11 g (10 mmol) of 2-(3-chloropropyl)-1,2,4-triazolo[4,3-a]pyridin-3-(2*H*)-one (**3a**)/2.56 g (10 mmol) of 2-(3-bromopropyl)-1,2,4-triazolo[4,3-a]pyridin-3-(2*H*)-one (**3b**), 2.33 g (10 mmol) of 1-(3-chlorophenyl) piperazine hydrochloride (**4**), 320 mg (1 mmol) of TBAB, 4.14 g (30 mmol) of K_2_CO_3_, and the appropriate amount of acetonitrile (8, 3, or 1 cm^3^)/DMF (6, 4, or 2 cm^3^)/water (50 cm^3^), were placed in a conical flask, after which the mixture was subjected to microwave radiation. The progress of the reaction was monitored by TLC (eluent chloroform–methanol 9:1). The reaction times are summarized in Table 2. After the reaction, 50 cm^3^ water was added and the resulting product was filtered off on a Büchner funnel. The reaction yields are summarized in Table 2. After drying, the obtained trazodone was dissolved in acetone and a solution of 2M HCl in dioxane was added until acidic (universal indicator). The precipitated hydrochloride was filtered off on a Büchner funnel.

#### 3.5.2. Preparation of Trazodone According to Method I under Microwave Conditions (Magnum II Reactor; 600 W)

Firstly, 168 mg (8 mmol) of 2-(3-chloropropyl)-1,2,4-triazolo[4,3-a]pyridin-3-(2*H*)-one (**3a**), 186 mg (8 mmol) of 1-(3-chlorophenyl)piperazine hydrochloride (**4**), 25.8 mg (0.8 mmol) of TBAB, 331 mg (2.4 mmol) of K_2_CO_3_, and 0.28 cm^3^ of ACN were placed in a PTFE vessel, after which the mixture was subjected to microwave radiation under pressure of 1, 5, or 10 bar. The reaction yields are summarized in Table 2. After drying, the obtained trazodone was dissolved in acetone and a solution of 2M HCl in dioxane was added until acidic (universal indicator). The precipitated hydrochloride was filtered off on a Büchner funnel.

### 3.6. General Procedures for the Preparation of 1-(3-Chloropropyl)-4-(3-chlorophenyl)piperazine (***5***)

#### 3.6.1. Preparation of 1-(3-Chloropropyl)-4-(3-chlorophenyl)piperazine (**5**) under Microwave Radiation (Samsung M182DN; 300 W)

Firstly, 2.50 cm^3^ (26 mmol, 4.09 g) of 1-bromo-3-chloropropane (**2a**), 2.33 g (10 mmol) of 1-(3-chlorophenyl)piperazine hydrochloride (**4**), 320 mg (10 mmol) of TBAB, 4.14 g (30 mmol) of K_2_CO_3_, and 3 cm^3^ of acetonitrile/DMF were placed in a conical flask, after which the mixture was subjected to microwave radiation. The progress of the reaction was monitored by TLC (eluent chloroform–methanol 9:1). After the reaction, 50 cm^3^ of water was added and the resulting product was filtered off. After drying, obtained **5** was dissolved in acetone and a solution of 2M HCl in dioxane was added until acidic (universal indicator). The precipitated hydrochloride was filtered off on a Büchner funnel; R_f_ = 0.83. The reaction yields are summarized in Table 3.

#### 3.6.2. Preparation of 1-(3-Chloropropyl)-4-(3-chlorophenyl)piperazine (**5**) under Microwave Radiation (Magnum II Reactor; 600 W)

Firstly, 0.25 cm^3^ (2.6 mmol, 409 mg) of 1-bromo-3-chloropropane (**2a**) 233 mg (1 mmol) of 1-(3-chlorophenyl) piperazine hydrochloride (**4**), 32 mg (1 mmol) of TBAB, 414 mg of K_2_CO_3_ (3 mmol), and 0.3 cm^3^ of acetonitrile were added in a PTFE vessel, after which the mixture was subjected to microwave radiation. The progress of the reaction was monitored by TLC (eluent chloroform–methanol 9:1). After drying, obtained **5** was dissolved in acetone and a solution of 2M HCl in dioxane was added until acidic (universal indicator). The precipitated hydrochloride was filtered off on a Büchner funnel.

### 3.7. General Procedures for the Preparation of Trazodone According to Method II

#### 3.7.1. Preparation of Trazodone According to Method II under Microwave Conditions (Samsung M182DN; 300 W)

Firstly, 2.76 g (10 mmol) of 1-(3-chloropropyl)-4-(3-chlorophenyl)piperazine hydrochloride (**5**), 1.35 g (10 mmol) of 1,2,4-triazolo[4,3-a]pyridin-3-(2*H*)-one (**1**), 322 mg (1 mmol) of TBAB, 4.14 g (30 mmol) of K_2_CO_3_, and a corresponding amount of acetonitrile (2, 6, or 8 cm^3^) were placed in a conical flask, after which the mixture was subjected to microwave radiation. The progress of the reaction was monitored by TLC (eluent chloroform–methanol 9:1). The reaction times are summarized in Table 4. After the reaction, about 50 cm^3^ of water was added and the resulting product was filtered off on a Büchner funnel. The reaction yields are summarized in Table 4; R_f_ = 0.75.

#### 3.7.2. Preparation of Trazodone According to Method II under Microwave Conditions (Magnum II Reactor; 600 W)

Firstly, 276 mg (1 mmol) of 1-(3-chloropropyl)-4-(3-chlorophenyl)piperazine hydrochloride (**5**), 135 mg (1 mmol) of 1,2,4-triazolo [4,3-a] pyridin-3-(2*H*)-one (**1**), 32 mg (0.1 mmol) of TBAB, 414 mg (3 mmol) of K_2_CO_3_, and 0.2 cm^3^ of acetonitrile were placed in a PTFE vessel, after which the mixture was subjected to microwave radiation. The progress of the reaction was monitored by TLC (eluent chloroform–methanol 9:1). After the reaction, water was added and the resulting product was filtered off. After drying to solution 1-(3-chloropropyl)-4-(3-chloropheyl)piperazine (**5**) in acetone, a solution of HCl in dioxane was added until acidic. The precipitated hydrochloride was filtered off on a Büchner funnel.

### 3.8. General Procedure for the Preparation of Trazodone in One-Pot Synthesis

Firstly, 1.35 g (10 mmol) of 1,2,4-triazolo[4,3-a]pyridin-3-(2*H*)-one (**1**), 2.5 cm^3^ (26 mmol) of 1-bromo-3-chloropropane (**2a**), 322 mg (1 mmol) of TBAB, 8.28 g (60 mmol) of K_2_CO_3_, and 5 cm^3^ of acetonitrile were placed in a conical flask. The reactions were carried out under microwave radiation (Samsung M182DN; 300 W) for 50 s. After this time, 2.3 g (10 mmol) of 1-(3-chlorophenyl)piperazine hydrochloride (**4**) and 5 cm^3^ of acetonitrile were added to the reaction mixture. Reactions were carried out in the presence of microwave radiation for another 90 s. The progress of the reaction was monitored by TLC (eluent chloroform–methanol 9:1). After the reaction, 50 cm^3^ of water was added and the resulting product was filtered off on a Büchner funnel. Yield (Samsung M182DN; 300 W) 31%, yield (CEM Discover SP reactor; 100 W) 71%.

### 3.9. General Procedure for the Preparation of 2-(3-Bromohexyl)-1,2,4-triazolo[4,3-a]pyridin-3-(2H)-one (***8***)

Firstly, 1.35 g (10 mmol) of 1,2,4-triazolo [4,3-a] pyridin-3-(2*H*)-one (**1**), 4.6 cm^3^ (30 mmol) of 1,6-dibromohexane, 0.322 g (1 mmol) of TBAB, 4.14 g (30 mmol) of K_2_CO_3_, and 2.5 cm^3^ of acetonitrile were placed in a conical flask, after which the mixture was subjected to microwave radiation for 60 s. The progress of the reaction was monitored by TLC (eluent chloroform–methanol 9:1). After the reaction, 15 cm^3^ of water was added to the mixture and extracted with methylene chloride. The product was purified by column chromatography (eluent chloroform–methanol 9:1).

*2-(3-bromohexyl)-1,2,4-triazolo[4,3-a]pyridin-3-(2H)-one* (**8**): ^1^H NMR (400 MHz, CDCl_3_) δ 7.78 (ddd, *J* = 5.5, 3.3, 2.2 Hz, 1H, ArH), 7.15–7.07 (m, 2H, ArH), 6.51 (ddd, *J* = 7.2, 4.4, 3.0 Hz, 1H, ArH), 4.02 (t, *J* = 7.1 Hz, 2H, CHNCO), 3.42 (dd, *J* = 8.0, 5.6 Hz, 2H, CHBr), 1.89 (ddd, *J* = 10.3, 7.3, 3.4 Hz, 4H, CH_Aliph_), 1.55–1.48 (m, 2H, CH_Aliph_), 1.46–1.39 (m, 2H, CH_Aliph_). ^13^C NMR (101 MHz, CDCl_3_) δ 148.57 (Ar), 141.47 (Ar), 129.75 (Ar), 123.77 (Ar), 115.38 (Ar), 110.48 (Ar), 45.77 (C_Aliph_), 33.69 (C_Aliph_), 32.56 (C_Aliph_), 28.62 (C_Aliph_), 27.71 (C_Aliph_), 25.75 (C_Aliph_). HPLC 91% (t_R_ = 7.31), R_f_ = 0,96, yield = 79%, oil.

### 3.10. General Procedure for the Preparation Trazodone Derivatives

Firstly, 529 mg (2.5 mmol) of 2-(3-chloropropyl)-1,2,4-triazolo[4,3-a]pyridin-3-(2*H*)-one (**3a**)/745 mg (2.5 mmol) of 2-(3-bromohexyl)-1,2,4-triazolo[4,3-a]pyridin-3-(2*H*)-one (**8**), 2.5 mmol of the corresponding arylpiperazine hydrochloride (**9a–m**), 80 g (0.25 mmol) of TBAB, 1.04 g (7.5 mmol) of K_2_CO_3_, and 0.75 cm^3^ of acetonitrile were placed in a conical flask, after which the mixture was subjected to microwave radiation (Samsung M182DN; 300 W). The progress of the reaction was monitored by TLC (eluent chloroform–methanol 9:1). After the reaction, water was added and the resulting product was filtered off on a Büchner funnel. After drying, the obtained **10a–k** were dissolved in acetone and a solution of 2M HCl in dioxane was added until acidic (universal indicator). The precipitated hydrochloride was filtered off on a Büchner funnel.

*2-[3-[4-(2,3-dichlorophenyl)piperazin-1-yl]propyl]-[1,2,4]triazolo[4,3-a]pyridin-3-one hydrochloride* (**10a**): ^1^H NMR (300 MHz, CDC_l3_) δ 7.76 (d, *J* = 7.1 Hz, 1H, ArH), 7.17 (ddd, *J* = 17.0, 8.8, 3.0 Hz, 4H, 1H, ArH), 7.02 (d, *J* = 8.0 Hz, 1H, 1H, ArH), 6.54 (t, *J* = 6.5 Hz, 1H, 1H, ArH), 4.18 (t, *J* = 6.1 Hz, 2H, CHNCO), 3.67 (dd, *J* = 22.9, 11.2 Hz, 4H, CH_pip_), 3.37 (d, *J* = 13.5 Hz, 2H, CH_pip_), 3.17 (s, 2H, CH_pip_), 3.06 (d, *J* = 11.5 Hz, 2H, CH_aliph_), 2.60 (s, 2H, CH_aliph_).^13^C NMR (75 MHz, CDCl_3_) δ 149.07 (Ar), 148.89 (Ar), 142.14 (Ar), 134.44 (Ar), 130.61 (Ar), 128.09 (Ar), 127.82 (Ar), 126.45 (Ar), 123.92 (Ar), 119.52 (Ar), 115.58 (Ar), 111.10 (Ar), 55.54 (C_triaz_), 52.66 (C_pip_, C_pip_), 48.15 (C_pip_, C_pip_), 43.45 (C_pip_), 23.55 (C_aliph_). Fourier-transform (FT)-IR 3000 (C–H Ar, Str), 2954, 2850 (C-H_Aliph_, Str), 1704 (C=O, Str), 1650 (C=N, Str), 1500, 1450 (C=C, Str), 1350 (C–N, Str), 750 (C–Cl, Str). HPLC 97% (*t*_R_ = 4.33), *m*/*z* = 406,19, R_f_ = 0.87, yield = 25%, melting point (mp) = 225–230 °C.

*2-[3-[4-(2-fluorophenyl)piperazin-1-yl]propyl]-[1,2,4]triazolo[4,3-a]pyridin-3-one hydrochloride* (**10b**): ^1^H NMR (300 MHz, CDCl_3_) δ 7.76 (d, *J* = 6.9 Hz, 1H, ArH), 7.26 (s, 2H, ArH), 7.12 (d, *J* = 10.0 Hz, 4H, ArH), 6.54 (s, 1H, ArH), 4.18 (s, 2H, CHNCO), 4.03–3.91 (m, 2H, CH_pip_), 3.62 (s, 2H, CH_pip_), 3.58–3.49 (m, 2H, CH_pip_), 3.46–3.30 (m, 2H, CH_pip_), 3.26–3.16 (m, 2H, CH_aliph_), 2.66–2.53 (m, 2H, CH_aliph_). ^13^C NMR (75 MHz, dimethyl sulfoxide (DMSO)) δ 155.88 (Ar), 152.63 (Ar), 147.58 (Ar), 140.62 (Ar), 137.71(Ar) 130.04 (Ar), 122.79 (Ar), 119.02 (Ar), 115.72 (Ar), 115.45 (Ar), 114.47 (Ar), 110.31 (Ar), 52.42 (C_triaz_), 50.35 (C_pip_, C_pip_), 46.41 (C_pip_, C_pip_), 42.01 (C_pip_), 22.38 (C_aliph_). FT-IR 3000 (C–H Ar, Str), 2946, 2852 (C–H_Aliph_, Str), 1711 (C=O, Str), 1639 (C=N, Str), 1574, 1451 (C=C, Str), 1355 (C–N, Str), 1108 (C–F, Str). HPLC 99% (*t*_R_ = 3.38), *m*/*z* = 356.21; R_f_ = 0.70, yield = 30%, mp = 146–150 °C.

*2-[3-(4-pyrimidin-2-ylpiperazin-1-yl)propyl]-[1,2,4]triazolo[4,3-a]pyridin-3-one hydrochloride* (**10c**): ^1^H NMR (300 MHz, CDCl_3_) δ 7.76 (d, *J* = 7.1 Hz, 1H, ArH), 7.26–7.07 (m, 4H, ArH), 7.02 (dd, *J* = 7.8, 1.7 Hz, 1H, ArH), 6.57–6.50 (m, 1H, ArH), 4.18 (t, *J* = 6.1 Hz, 2H, CHNCO), 3.67 (dd, *J* = 22.2, 11.3 Hz, 4H, CH_pip_), 3.37 (d, *J* = 12.9 Hz, 2H, CH_pip_), 3.25–3.15 (m, 2H, CH_pip_), 3.06 (d, *J* = 11.3 Hz, 2H, CH_aliph_), 2.59 (t, *J* = 14.4 Hz, 2H, CH_aliph_). ^13^C NMR (75 MHz, CDCl_3_) δ 148.85 (Ar), 148.67 (Ar), 141.93 (Ar), 130.41 (Ar), 127.89 (Ar, Ar) 126.23 (Ar), 119.32 (Ar), 115.37 (Ar), 110.90 (Ar), 55.32(C_triaz_), 52.46 (C_pip_, C_pip_), 47.94 (C_pip_, C_pip_), 43.24 (C_pip_), 23.34 (C_aliph_). FT-IR 2990 (C-H Ar, Str), 2946, 2850 (C–H_Aliph_, Str), 1706 (C=O, Str), 1636 (C=N, Str), 1601, 1459 (C=C, Str), 1355 (C–N, Str). HPLC 99% (*t*_R_ = 4.32), *m*/*z* = 340.46; R_f_ = 0.68, yield = 34%, mp = 235–240 °C.

*2-[3-[4-(2-ethoxyphenyl)piperazin-1-yl]propyl]-[1,2,4]triazolo[4,3-a]pyridin-3-one hydrochloride* (**10d**): ^1^H NMR (300 MHz, CDCl_3_) δ 7.76 (d, *J* = 7.1 Hz, 1H, ArH), 7.18–6.95 (m, 4H, ArH), 6.89 (dd, *J* = 17.3 7.4 Hz, 2H, ArH), 6.54 (t, *J* = 6.5 Hz, 1H, ArH), 4.17 (t, *J* = 6.1 Hz, 2H, CHNCO), 4.08 (q, *J* = 6.9 Hz, 2H, OCH), 3.65–3.53 (m, 4H, CH_pip_), 3.16 (s, 4H, CH_pip_), 2.60 (s, 2H, CH_aliph_), 1.64 (s, 2H, CH_aliph_), 1.59 (s, 2H, CH_aliph_). ^13^C NMR (75 MHz, CDCl_3_) δ 151.66 (Ar), 148.87 (Ar), 142.17 (Ar, Ar), 130.62 (Ar), 123.92 (Ar, Ar), 121.47 (Ar), 115.59 (Ar, Ar), 113.05 (Ar), 111.11 (Ar), 64.37 (C_oxy_), 55.39 (C_triaz_), 51.37 (C_pip_, C_pip_), 48.08 (C_pip_, C_pip_), 43.41 (C_pip_), 23.53 (C_aliph_), FT-IR 3001 (C–H Ar, Str), 2941, 2858 (C–H_Aliph_, Str), 1708 (C=O, Str), 1611 (C=N, Str), 1531, 1458 (C=C, Str), 1349 (C–N, Str), 1260, 1021 (C–O, Str). HPLC 98% (*t*_R_ = 3.73), *m*/*z* = 382.27; R_f_ = 0.66, yield = 33%, mp = 104–111 °C.

*2-[6-[4-(3-chlorophenyl)piperazin-1-yl]hexyl]-[1,2,4]triazolo[4,3-a]pyridin-3-one hydrochloride* (**10e**): ^1^H NMR (300 MHz, CDCl_3_) δ 7.76 (d, *J* = 7.1 Hz, 1H, ArH), 7.61 (s, 1H, ArH), 7.49 (d, *J* = 8.2 Hz, 1H, ArH), 7.39 (d, *J* = 8.2 Hz, 1H, ArH), 7.31 (d, *J* = 8.3 Hz, 1H, ArH), 7.15–7.05 (m, 2H, ArH), 6.51 (dt, *J* = 7.3, 3.7 Hz, 1H, ArH), 4.45 (d, *J* = 11.4 Hz, 2H, CHNCO), 4.02 (t, *J* = 6.7 Hz, 2H, CH_pip_), 3.83 (s, 2H, CH_pip_), 3.73–3.63 (m, 4H, CH_pip_), 3.11 (s, 2H, CH_aliph_), 2.00–1.81 (m, 4H, CH_aliph_), 1.46 (s, 4H, CH_aliph_). ^13^C NMR (75 MHz, CDCl_3_) δ 148.62(Ar), 146.74 (Ar), 141.56 (Ar), 135.80 (Ar), 131.06 (Ar), 129.92 (Ar), 125.69 (Ar), 123.72 (Ar), 119.31 (Ar), 116.98 (Ar), 115.37 (Ar), 110.65 (Ar), 57.28 (C_triaz_), 50.21 (C_pip,_ C_pip_), 48.35 (C_pip_, C_pip_), 45.33 (C_pip_), 28.32 (C_aliph_), 26.06 (C_aliph_), 25.67 (C_aliph_), 23.39 (C_aliph_). FT-IR 2981 (C–H Ar, Str), 2935, 2851 (C–H_Aliph_, Str), 1703 (C=O, Str), 1639 (C=N, Str), 1593, 1451 (C=C, Str), 1354 (C–N, Str), 734 (C–Cl, Str). HPLC 97% (*t*_R_ = 4.23), *m*/*z* = 414.30, R_f_ = 0.66, yield = 47% (Samsung), yield = 58 (CEM Discover SP reactor), mp = 178–183 °C.

*2-[6-[4-(2-chlorophenyl)piperazin-1-yl]hexyl]-[1,2,4]triazolo[4,3-a]pyridin-3-one hydrochloride* (**10f**): ^1^H NMR (300 MHz, DMSO) δ 8.44 (d, *J* = 4.8 Hz 1H, ArH), 8.33–8.17 (m, 2H, ArH), 7.93–7.82 (m, 1H, ArH), 7.63 (dd, *J* = 11.7, 6.5 Hz, 2H, ArH), 7.08 (dd, *J* = 13.6, 6.4 Hz, 1H, ArH), 6.75 (t, *J* = 4.7 Hz, 1H, ArH), 3.84 (t, *J* = 7.3 Hz, 2H, CHNCO), 3.58 (s, 8H, CH_pip_), 3.04 (m, 2H, CH_aliph_), 1.84 (m, 4H, CH_aliph_), 1.52 (m, 4H, CH_aliph_). ^13^C NMR (101 MHz, DMSO) δ 161.06 (Ar), 158.58 (Ar), 136.22 (Ar), 132.01 (Ar), 130.58 (Ar), 130.23 (C Ar), 129.15 (Ar), 120.70 (Ar), 118.62 (Ar), 118.54(Ar), 111.66 (Ar), 104.35 (Ar), 55.87 (C_triaz_), 50.74 (C_pip_, C_pip_), 42.71 (C_pip_, C_pip_), 41.81 (C_pip_), 27.98 (C_aliph_), 27.82 (C_aliph_), 27.61 (C_aliph_), 26.22 (C_aliph_). FT-IR 3008 (C–H Ar, Str), 2942, 2856 (C–H_Aliph_, Str), 1738 (C=O, Str), 1616 (C=N, Str), 1538, 1456 (C=C, Str), 1349 (C–N, Str), 738 (C–Cl, Str). HPLC 92% (*t*_R_ = 4.86), *m*/*z* = 452.19; R_f_ = 0.67, yield = 45%, mp = 145–148 °C.

*2-[6-[4-(4-chlorophenyl)piperazin-1-yl]hexyl]-[1,2,4]triazolo[4,3-a]pyridin-3-one hydrochloride* (**10g**): ^1^H NMR (300 MHz, CDCl_3_) δ 7.85 (d, *J* = 8.8 Hz, 2H, ArH), 7.77 (d, *J* = 7.1 Hz, 1H, ArH), 7.51 (d, *J* = 8.9 Hz, 2H, ArH), 7.13–7.09 (m, 2H, ArH), 6.52 (dd, *J* = 7.2, 3.6 Hz, 1H, ArH), 4.78 (m, 2H, CHNCO), 4.21 (m, 2H, CH_pip_), 4.04 (t, *J* = 6.8 Hz, 2H, CH_pip_), 3.75–3.56 (m, 4H, CH_pip_), 3.15 (m, 2H, CH_aliph_), 2.03–1.81 (m, 4H, CH_aliph_), 1.45 (m, 4H, CH_aliph_). ^13^C NMR (101 MHz, DMSO) δ 148.89 (Ar), 148.39 (Ar), 141.35 (Ar), 130.85 (Ar), 129.27 (Ar, Ar), 124.30 (Ar) 124.00 (Ar), 117.93 (Ar, Ar), 115.44 (Ar), 111.30 (Ar), 55.69 (C_triaz_), 50.79 (C_pip_, C_pip_), 45.64 (C_pip_, C_pip_), 45.26 (C_pip_), 28.44 (C_aliph_), 25.96 (C_aliph_), 25.87 (C_aliph_), 23.29 (C_aliph_). FT-IR 3034 (C–H Ar, Str), 2934, 2860 (C–H_Aliph_, Str), 1708 (C=O, Str), 1637 (C=N, Str), 1539, 1482 (C=C, Str), 1354 (C–N, Str), 755 (C–Cl, Str). HPLC 98% (*t*_R_ = 1.63); R_f_ = 0.50, yield = 44%, mp = 163–167 °C.

*2-[6-[4-(3,4-dichlorophenyl)piperazin-1-yl]hexyl]-[1,2,4]triazolo[4,3-a]pyridin-3-one hydrochloride* (**10h**): ^1^H NMR (300 MHz, DMSO) δ 7.84 (d, *J* = 7.1 Hz, 1H, ArH), 7.45 (d, *J* = 8.9 Hz, 2H, ArH), 7.23 (dd, *J* = 5.9, 3.4 Hz, 2H, ArH), 7.00 (dd, *J* = 9.1, 2.8 Hz, 1H, ArH), 6.66–6.58 (m, 1H, ArH), 4.67 (s, 2H, CHNCO), 3.89 (dd, *J* = 12.3, 5.2 Hz, 4H, CH_pip_), 3.60–3.47 (m, 4H, CH_pip_), 3.16 (m, 2H, CH_aliph_), 1.75 (m, 4H, CH_aliph_), 1.32 (m, 4H, CH_aliph_). ^13^C NMR (101 MHz, DMSO) δ 149.77 (Ar), 148.38 (Ar), 141.35 (Ar), 132.11 (Ar), 131.10 (Ar), 130.84 (Ar), 124.29 (Ar), 121.22 (Ar), 117.44 (Ar), 116.29 (Ar), 115.44 (Ar), 111.30 (Ar), 55.73 (C_triaz_), 50.54 (C_pip_), 45.71–44.67 (C_pip,_ C_pip_), 42.66 (C_pip,_ C_pip_), 32.66 (C_pip_), 28.44 (C_aliph_), 25.68 (C_aliph_), 23.34 (C_aliph_), 22.55(C_aliph_). FT-IR 3016 (C–H Ar, Str), 2949, 2860 (C–H_Aliph_, Str), 1738 (C=O, Str), 1642 (C=N, Str), 1542 (C=C, Str), 1365 (C–N, Str), 757 (C–Cl, Str)**.** HPLC 95% (*t*_R_ = 4.79), *m*/*z* = 448.20; R_f_ = 0.54, yield = 45%, mp = 116–120 °C.

*2-[6-[4-(2-fluorophenyl)piperazin-1-yl]hexyl]-[1,2,4]triazolo[4,3-a]pyridin-3-one hydrochloride* (**10i**): ^1^H NMR (400 MHz, DMSO) δ 7.85 (dt, *J* = 7.1, 1.1 Hz, 1H, ArH), 7.20–7.03 (m, 5H, ArH), 6.62 (ddd, *J* = 7.2, 5.0, 2.4 Hz, 1H, ArH), 3.90 (t, *J* = 6.9 Hz, 2H, CHNCO), 3.55 (t, *J* = 9.5 Hz, 2H, CH_pip_), 3.46 (d, *J* = 11.1 Hz, 2H, CH_pip_), 3.24–3.15 (m, 4H, CH_pip_), 3.08 (dd, *J* = 10.9, 5.5 Hz, 2H, CH_aliph_), 1.78–1.69 (m, 4H, CH_aliph_), 1.35–1.29 (m, 4H, CH_aliph_). ^13^C NMR (101 MHz, DMSO) δ 154.21–153.60 (Ar), 148.39 (Ar), 141.35 (Ar), 138.74 (Ar), 130.84 (Ar), 125.46 (Ar), 124.31 (Ar), 120.01 (Ar), 116.71 (Ar), 116.51 (v), 115.45 (Ar), 111.30 (Ar), 55.79 (C_triaz_), 51.22 (C_pip_, C_pip_), 47.41 (C_pip_, C_pip_), 45.26 (C_pip_), 28.44 (C_aliph_), 26.05 (C_aliph_), 25.89 (C_aliph_), 23.29 (C_aliph_). FT-IR 3021 (C–H Ar, Str), 2936, 2850 (C–H_Aliph_, Str), 1713 (C=O, Str), 1635 (C=N, Str), 1541, 1500 (C=C, Str), 1355 (C–N, Str), 1141 (C–F, Str). HPLC 94% (*t*_R_ = 1.46), R_f_ = 0.51, yield = 45%, mp = 183–187 °C.

*2-[6-[4-(2-methoxyphenyl)piperazin-1-yl]hexyl]-[1,2,4]triazolo[4,3-a]pyridin-3-one hydrochloride* (**10j**): ^1^H NMR (300 MHz, CDCl_3_) δ 8.26 (d, *J* = 6.4 Hz, 1H, ArH), 7.77 (d, *J* = 7.0 Hz, 1H, ArH), 7.55–7.42 (m, 1H, ArH), 7.08 (dd, *J* = 14.9, 6.3 Hz, 4H, ArH), 6.50 (m, 1H, ArH), 5.16 (m, 3H, OCH), 4.46 (m, 2H, CHNCO), 4.13–3.95 (m, 4H, CH_pip_), 3.66–3.52 (m, 4H, CH_pip_), 3.14 (s, 2H, CH_Aliph_), 2.02–1.76 (m, 4H, CH_aliph_), 1.45 (m, 4H, CH_aliph_). ^13^C NMR (101 MHz, DMSO) δ 152.27 (Ar), 148.39 (Ar), 141.36 (Ar), 139.75 (Ar), 130.85 (Ar), 124.30 (Ar), 124.00 (Ar), 121.30 (Ar), 118.72 (Ar), 115.45 (Ar), 112.41 (Ar), 111.31 (Ar), 55.84 (C_oxy_), 51.45 (C_triaz_), 47.32 (C_pip_), 45.75 (C_pip_), 45.27 (C_pip,_ C_pip_), 32.18 (C_pip_), 28.44 (C_aliph_), 26.56 (C_aliph_), 26.16 (C_aliph_), 25.79 (C_aliph_), 23.30 (C_aliph_). FT-IR 3016 (C–H Ar, Str), 2942, 2860 (C–H_Aliph_, Str), 1708 (C=O, Str), 1640 (C=N, Str), 1540, 1448 (C=C, Str), 1366 (C–N, Str), 1261, 1020 (C–O, Str). HPLC 95% (*t*_R_ = 3.66), R_f_ = 0.45, yield = 32%, mp = 140–141 °C.

*2-[6-[4-(3-methoxyphenyl)piperazin-1-yl]hexyl]-[1,2,4]triazolo[4,3-a]pyridin-3-one hydrochloride* (**10k**): ^1^H NMR (300 MHz, CDCl_3_) δ 7.77 (d, *J* = 7.1 Hz, 1H, ArH), 7.49 (s, 1H, ArH), 7.42 (d, *J* = 7.5 Hz, 2H, ArH), 7.11 (d, *J* = 4.1 Hz, 2H, ArH), 7.01 (d, *J* = 7.4 Hz, 1H, ArH), 6.51 (dd, *J* = 7.2, 3.8 Hz, 1H, ArH), 4.78 (s, 3H, OCH), 4.29 (s, 2H, CHNCO), 4.03 (t, *J* = 6.8 Hz, 2H, CH_pip_), 3.86 (s, 2H, CH_pip_), 3.66 (d, *J* = 10.9 Hz, 4H, CH_pip_), 3.15 (s, 2H, CH_aliph_), 1.91 (dd, *J* = 14.3, 7.3 Hz, 4H, CH_aliph_), 1.48 (d, *J* = 24.1 Hz, 4H, CH_aliph_). ^13^C NMR (101 MHz, DMSO) δ 150.21 (Ar), 140.44 (Ar, Ar), 139.99 (Ar), 129.15 (Ar), 120.10 (Ar, Ar), 121.16 (Ar), 119.89 (Ar), 114.42 (Ar), 112.11 (Ar), 111.11 (Ar), 55.44 (C_oxy_), 50.42 (C_triaz_), 47.39 (C_pip_), 45.85 (C_pip,_ C_pip_), 32.21 (C_pip_), 28.41 (C_aliph_), 27.14 (C_aliph_), 28.12 (C_aliph_), 25.67 (C_aliph_), 23.31 (C_aliph_). FT-IR 2982 (C–H Ar, Str), 2938, 2861 (C–H_Aliph_, Str), 1640 (C=O, Str), 1615 (C=N, Str), 1541, 1491 (C=C, Str), 1356 (C–N, Str), 1271, 1027 (C–O, Str). HPLC 100% (*t*_R_ = 3.88), R_f_ = 0.54, yield = 30%, mp = 137–138 °C.

*2-[6-[4-(4-methoxyphenyl)piperazin-1-yl]hexyl]-[1,2,4]triazolo[4,3-a]pyridin-3-one hydrochloride*: (**10l**) ^1^H NMR (300 MHz, CDCl_3_) δ 7.86 (d, *J* = 9.2 Hz, 2H, ArH), 7.76 (m, *J* = 7.0 Hz, 1H, ArH), 7.10 (d, *J* = 2.9 Hz, 2H, ArH), 7.00 (d, *J* = 9.1 Hz, 2H, ArH), 6.51 (m, 1H, ArH), 4.80 (m, 3H, OCH), 4.45–4.26 (m, 2H, CHNCO), 4.04 (t, *J* = 6.8 Hz, 2H, CH_pip_), 3.84 (m, 2H, CH_pip_), 3.62 (m, 4H, CH_pip_), 3.13 (s, 2H, CH_aliph_), 1.91 (m, 4H, CH_aliph_), 1.73 (m, 4H, CH_aliph_). ^13^C NMR (101 MHz, DMSO) δ 154.37 (Ar), 148.39 (Ar), 143.82 (Ar), 141.36 (Ar), 130.85 (Ar), 124.30 (Ar), 118.65 (Ar), 115.45 (Ar, Ar) 114.88 (Ar, Ar), 111.31 (Ar), 56.98 (C_oxy_), 55.70 (C_triaz_), 51.03 (C_pip_, C_pip_), 47.31 (C_pip_, C_pip_), 45.27 (C_pip_), 28.44 (C_aliph_), 26.50–25.58 (C_aliph_), 23.31 (C_aliph_), 13.96 (C_aliph_). FT-IR 3002 (C–H Ar, Str), 2939, 2840 (C–H_Aliph_, Str), 1708 (C=O, Str), 1640 (C=N, Str), 1541, 1509 (C=C, Str), 1374 (C–N, Str), 1260, 1025 (C–O, Str). HPLC 92% (*t*_R_ = 1.40), R_f_ = 0.65, yield = 27%, mp = 144–146 °C.

*2-[6-[4-(2-ethoxyphenyl)piperazin-1-yl]hexyl]-[1,2,4]triazolo[4,3-a]pyridin-3-one hydrochloride* (**10m**): ^1^H NMR (300 MHz, CDCl_3_) δ 8.20–8.15 (s, 1H, ArH), 7.77 (d, *J* = 7.1 Hz, 1H, ArH), 7.43 (m, 1H, ArH), 7.08 (dd, *J* = 15.7, 4.7 Hz, 4H, ArH), 6.51 (s, 1H, ArH), 4.96 (m, 2H, OCH), 4.45 (m, 2H, CHNCO), 4.33 (t, *J* = 7.3 Hz, 2H, CH_pip_), 4.02 (t, *J* = 6.9 Hz, 2H, CH_pip_), 3.61 (t, *J* = 13.0 Hz, 4H, CH_pip_), 3.13 (s, 2H, CH_aliph_), 1.89 (m, 4H, CH_aliph_), 1.64 (t, *J* = 7.0 Hz, 3H, CH_aliph_), 1.44 (m, 4H, CH_aliph_). ^13^C NMR (101 MHz, DMSO) δ 155.67 (Ar), 147.23 (Ar), 144.81 (Ar), 139.45 (Ar), 137.84 (Ar), 120.12 (Ar), 120.01 (Ar), 116.42 (Ar, Ar), 112.98 (Ar, Ar), 110.31 (Ar), 58.91 (C_oxy_), 55.74 (C_triaz_), 50.04 (C_pip_, C_pip_), 48.21 (C_pip_, C_pip_), 44.18 (C_pip_), 29.60 (C_aliph_), 26.12 (C_aliph_), 21.13 (C_aliph_), 13.90 (C_aliph_), 13.40 (C_aliph_). FT-IR 2990 (C–H Ar, Str), 2936; 2860 (C–H_Aliph_, Str), 1701 (C=O, Str), 1636 (C=N, Str), 1541, 1491 (C=C, Str), 1356 (C–N, Str), 1251, 1040 (C–O, Str). HPLC 97% (*t*_R_ = 1.63), R_f_ = 0.46, yield = 31%, mp = 143–145 °C.

*2-[6-[4-(2-pyridyl)piperazin-1-yl]hexyl]-[1,2,4]triazolo[4,3-a]pyridin-3-one hydrochloride* (**10n**): ^1^H NMR (300 MHz, DMSO) δ 8.14 (d, *J* = 4.1 Hz, 1H, ArH), 7.83 (t, *J* = 7.0 Hz, 2H, ArH), 7.22 (d, *J* = 3.2 Hz, 2H, ArH), 7.15 (d, *J* = 8.9 Hz, 1H, ArH), 6.88 (s, 1H, ArH), 6.67–6.57 (m, 1H, ArH), 4.40 (d, *J* = 13.8 Hz, 2H, CHNCO), 3.88 (dd, *J* = 16.3, 9.5 Hz, 2H, CH_pip_), 3.58 (d, *J* = 11.6 Hz, 2H, CH_pip_), 3.37 (d, *J* = 14.1 Hz, 2H, CH_pip_), 3.22 (s, 2H, CH_pip_), 3.08 (s, 2H, CH_aliph_), 1.75 (d, *J* = 6.8 Hz, 4H, CH_aliph_), 1.28 (d, *J* = 27.2 Hz, 4H, CH_aliph_). ^13^C NMR (101 MHz, DMSO) δ 148.38 (Ar), 141.35 (Ar), 130.85 (Ar, Ar), 124.30 (Ar, Ar), 115.44 (Ar), 114.27 (Ar, Ar), 114.16 (Ar), 111.31 (Ar), 55.80 (C_triaz_), 50.29 (C_pip_), 45.27 (C_pip_), 43.34 (C_pip_), 42.34 (C_pip_), 28.44 (C_aliph_), 26.02 (C_aliph_), 25.88 (C_aliph_), 23.28 (C_aliph_). FT-IR 2970 (C–H Ar, Str), 2936, 2861 (C–H_Aliph_, Str), 1701 (C=O, Str), 1633 (C=N, Str), 1536, 1495 (C=C, Str), 1365 (C–N, Str). HPLC 98% (*t*_R_ = 2.32), *m*/*z* = 381.27, R_f_ = 0.48, yield = 70%, mp = 175–178 °C.

*2-[6-(4-pyrimidin-2-ylpiperazin-1-yl)hexyl]-[1,2,4]triazolo[4,3-a]pyridin-3-one hydrochloride* (**10o**): ^1^H NMR (300 MHz, CDCl_3_) δ 8.61 (d, *J* = 5.2 Hz, 1H, ArH), 8.29 (s, 1H, ArH), 7.76 (s, 1H, ArH), 7.10 (s, 2H, ArH), 6.99 (s, 1H, ArH), 6.54–6.50 (m, 1H, ArH), 4.28–4.21 (m, 2H, CHNCO), 4.01 (s, 4H, CH_pip_), 3.70 (s, 4H, CH_pip_), 3.07–3.00 (m, 2H, CH_aliph_), 1.95–1.81 (m, 4H, CH_aliph_), 1.45–1.38 (m, 4H, CH_Aliph_). ^13^C NMR (75 MHz, CDCl_3_) δ 157.67 (Ar), 148.81 (Ar), 141.73 (Ar), 130.08 (Ar), 123.96 (Ar), 115.59 (Ar), 111.38 (Ar), 110.82 (Ar), 109.87 (Ar), 57.58 (C_triaz_), 51.54 (C_pip_, C_pip_), 45.57 (C_pip_, C_pip_), 41.78 (C_pip_), 29.56 (C_aliph_), 28.11 (C_aliph_), 26.16 (C_aliph_), 23.54 (C_aliph_). FT-IR 3023 (C–H Ar, Str), 2937, 2857 (C–H_Aliph_, Str), 1700 (C=O, Str), 1616 (C=N, Str), 1540 (C=C, Str), 1350 (C–N, Str). HPLC 90%, (*t*_R_ = 1.42), R_f_ = 0.35, yield = 45% (Samsung), yield = 61% (CEM Discover SP reactor), mp = 146–148 °C.

*2-[6-(4-phenylpiperazin-1-yl)hexyl]-[1,2,4]triazolo[4,3-a]pyridin-3-one hydrochloride* (**10p**): ^1^H NMR (300 MHz, DMSO) δ 7.84 (d, *J* = 7.0 Hz, 1H, ArH), 7.25 (dd, *J* = 14.6, 5.8 Hz, 4H, ArH), 7.00 (d, *J* = 8.1 Hz, 2H, ArH), 6.86 (t, *J* = 7.2 Hz, 1H, ArH), 6.61 (dd, *J* = 8.4, 5.8 Hz, 1H, ArH), 3.77 (t, *J* = 12.0 Hz, 2H, CHNCO), 3.64–3.35 (m, 4H, CH_pip_), 3.16 (dd, *J* = 22.3, 9.6 Hz, 4H, CH_pip_), 3.06 (m, 2H, CH_aliph_), 1.74 (m, 4H, CH_aliph_), 1.32 (m, 4H, CH_aliph_). FT-IR 2987 (C–H Ar, Str), 2937, 2861 (C–H_Aliph_, Str), 1693 (C=O, Str), 1639 (C=N, Str), 1541, 1491 (C=C, Str), 1375 (C–N, Str). HPLC 90% (*t*_R_ = 3.69), *m*/*z* = 380.27, R_f_ = 0.58, yield = 55%, mp = 133–137 °C. 

*2-[6-[4-(2-phenylphenyl)piperazin-1-yl]hexyl]-[1,2,4]triazolo[4,3-a]pyridin-3-one hydrochloride* (**10r**): ^1^H NMR (300 MHz, CDCl_3_) δ 7.76 (d, *J* = 7.2 Hz, CH-, 1H, ArH), 7.52 (d, *J* = 7.2 Hz, 2H, ArH), 7.42 (t, *J* = 7.5 Hz, 2H, ArH), 7.37–7.29 (m, 2H, ArH), 7.24–7.22 (m, 1H, ArH), 7.21–7.13 (m, 2H, ArH), 7.09 (d, *J* = 3.2 Hz, 2H, ArH), 6.54–6.46 (m, 1H, ArH), 3.99 (t, *J* = 7.0 Hz, 2H, CHNCO), 3.53 (t, *J* = 12.0 Hz, 2H, CH_pip_), 3.35 (d, *J* = 10.9 Hz, 2H, CH_pip_), 3.10 (d, *J* = 13.4 Hz, 2H, CH_pip_), 2.86 (m, 2H, CH_pip_), 2.69 (m, 2H, CH_aliph_), 1.81 (m, 4H, CH_aliph_), 1.39 (m, 4H, CH_aliph_). ^13^C NMR (101 MHz, DMSO) δ 148.71 (Ar), 148.37 (Ar), 141.34 (Ar), 140.59 (Ar), 134.47 (Ar), 131.74 (Ar), 130.84 (Ar), 129.03 (Ar, Ar), 128.73 (Ar, Ar), 127.55 (Ar), 124.29 (Ar), 123.96 (Ar), 119.01 (Ar, Ar), 115.44 (Ar), 111.30 (Ar), 55.75 (C_triaz_), 51.25 (C_pip_), 47.76 (C_pip,_ C_pip_), 45.25 (C_pip,_ C_pip_), 28.42 (C_aliph_), 26.01 (C_aliph_), 25.84 (C_aliph_), 23.25 (C_aliph_). FT-IR 3023 (C–H Ar, Str), 2937, 2858 (C–H_Aliph_, Str), 1702 (C=O, Str), 1637 (C=N, Str), 1541, 1500 (C=C, Str), 1351 (C–N, Str). HPLC 95% (*t*_R_ = 4.99), *m*/*z* = 456.94, R_f_ = 0.65, yield = 61% (Samsung), yield = 66% (CEM Discover SP reactor), oil.

## 4. Conclusions

As part of the research, 17 new trazodone derivatives were obtained, which were tested for their activity toward the 5-HT_1A_ receptor, which is an important hold of drugs used in the diseases of the central nervous system. All ligands, as well as substrates for them, were obtained by means of the new synthesis method in the presence of potassium carbonate as a reaction medium under microwave radiation. The in vitro studies showed that, in the obtained group of ligands, there are selective ligands for the 5-H_1A_ receptor (**10e**, **10g**, **10h**, **10i**, **10m**, and **10p**). Among the ligands obtained, those having a hexyl chain, as compared with a propyl chain, have a higher incremental activity. It is very interesting that the elongation in the trazodone of the alkyl chain by three carbon atoms changes the activity profile from the 5-HT_2A_ ligand (**trazodone**, 5-HT_1A_ *K*_i_ = 78, 5-HT_2A_ *K*_i_ = 16) to 5-HT_1A_ (**10e**, 5-HT_1A_ *K*_i_ = 16, 5-HT_2A_ *K*_i_ = 342). This change in the activity was evaluated in the molecular modeling procedure and additionally supported with crystallographic studies. A very interesting pharmacological profile is shown by the compound **10r**, which has the properties of a dual 5-HT_1A_/_7_ ligand (5-HT_1A_ *K*_i_ = 20, 5-HT_7_ *K*_i_ = 19). Bearing in mind all of the above, we are continuing the research on the activity of these ligands (**10e**, **10r**). Currently, we are conducting in vivo studies, the aim of which is to confirm their antidepressant activity.

## 5. Patents

Some of the results concerning the synthesis of trazodone are presented in “Method for the preparation trazodone” PCT/PL2018/000024, 6 March 2018.

## Figures and Tables

**Table 1:** Synthesis 2-(3-halopropyl)[1,2,4]triazolo[4,3-a]pyridin-3(2*H*)-one (**3a/b**)—0.01 mol of 1,2,4-triazolo[4,3-a]pyridin-3-(2*H*)-one (**1**), 0.026 mol of 1-bromo-3-chloropropane (**2a**)/0.026 mol 1,3-dibromopropane (**2b**), 0.001 mol of tetrabutylammonium bromide (TBAB), 0.03 mol of K_2_CO_3_. DMF—dimethylformamide; ACN—acetonitrile.

No.	Solvent	Amount of Solvent (cm^3^)	X	Time (s)	Yield (%)
1	DMF	5	Cl	50	24
2	ACN	6	Cl	50	83
3	ACN	3	Cl	80	92
4	ACN	0.75	Cl	50	90
5 *	ACN	0.075	Br	120	81
6	ACN	0.75	Br	60	73
7	ACN	0.4	Cl	60	82
8	-	-	Cl	80	81

* Reaction was carried out in a MAGNUM II reactor. Molar amounts of the reactants were reduced 10-fold.

**Table 2:** Synthesis of trazodone with method I—10 mmol of 2-(3-chloropropyl)-1,2,4-triazolo[4,3-a]pyridin-3-(2*H*)-one (**3a**) /10 mmol of 2-(3-bromopropyl)-1,2,4-triazolo[4,3-a]pyridin-3-(2*H*)-one (**3b**), 10 mmol of 1-(3-chlorophenyl) piperazine hydrochloride (**4**), 1 mmol of TBAB, 30 mmol of K_2_CO_3_. PTC— phase transfer catalyst; DABCO—1,4-diazabicyclo[2.2.2]octane.

No.	Solvent	Amount of Solvent (cm^3^)	PTC	X	Time (s)	Yield (%)
1	DMF	4	TBAB	Br	60	91
2	DMF	2	TBAB	Br	60	98
3	DMF	2	TEAC	Br	120	90
4	DMF	2	DABCO	Br	120	83
5	-	-	TBAB	Br	100	69
6	-	-	TBAB	Br	300	82
7	ACN	3	TBAB	Br	60	78
8	ACN	8	TBAB	Cl	60	90
9	ACN	3	TBAB	Cl	60	86
10	ACN	1	TBAB	Cl	80	89
11	-	-	TBAB	Cl	100	76
12	DMF	6	TBAB	Cl	60	50
13	H_2_O	50	TBAB	Cl	60	51
14 *	ACN	0.28	TBAB	Cl	120 (1 bar)	86
15 *	ACN	0.28	TBAB	Cl	120 (5 bar)	86
16 *	ACN	0.28	TBAB	Cl	120 (10 bar)	65

* Reaction was carried out in a MAGNUM II reactor; reactions were carried out in a closed vessel with a given pressure 1, 5, or 10 bar. Molar quantities of reactants—8 mmol of 2-(3-chloropropyl)-1,2,4-triazolo[4,3-a]pyridin-3-(2*H*)-one (**3a**), 8 mmol of 1-(3-chlorophenyl) piperazine hydrochloride (**4**), 0.8 mmol of TBAB, 2.4 mmol of K_2_CO_3_.

**Table 3:** Synthesis 1-(3-chloropropyl)-4-(3-chlorophenyl)piperazine (**5**)—26 mmol of 1-bromo-3-chloropropane (**2a**), 10 mmol of 1-(3-chlorophenyl) piperazine hydrochloride (**4**), 10 mmol of TBAB, 30 mmol of K_2_CO_3_.

No.	Solvent	Amount of Solvent (cm^3^)	Time (s)	Yield (%)
1	DMF	3	120	62
2	ACN	3	40	88
3 *	ACN	0.3	120	83

* Reaction was carried out in a MAGNUM II reactor. Molar amounts of the reactants were reduced 10-fold.

**Table 4:** Synthesis of trazodone with method II—10 mmol of 1-(3-chloropropyl)-4-(3-chlorophenyl)piperazine hydrochloride (**5**), (10 mmol) of 1,2,4-triazolo[4,3-a]pyridin-3-(2*H*)-one (**1**), 10 mmol of TBAB, 30 mmol of K_2_CO_3_.

No.	Solvent	Amount of Solvent (cm^3^)	Time (s)	Yield (%)
1	ACN	8	80	92
2	ACN	6	60	73
3	ACN	6	80	82
4	ACN	2	80	92
5 *	ACN	0.2	120	77

* Reaction was carried out in a MAGNUM II reactor. Molar amounts of the reactants were reduced 10-fold.

**Table 5:** Analytical data of the library **10a**–**10k.** M.P.—melting point.

Entry	No.	*n*	R	Yield (%)	Purity* (%)	M.P. (°C)
1	**10a**	1	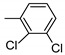	25	97	225–230
2	**10b**	1	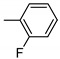	30	99	146–150
3	**10c**	1	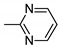	34	99	235–240
4	**10d**	1	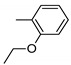	33	98	104–111
5	**10e**	4	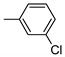	44	97	178–183
6	**10f**	4	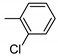	45	92	145–148
7	**10g**	4	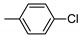	44	98	163–167
8	**10h**	4	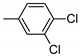	45	98	116–120
9	**10i**	4	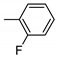	45	94	183–185
10	**10j**	4	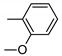	32	95	140–141
11	**10k**	4	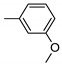	30	100	137–138
12	**10l**	4	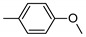	27	92	144–146
14	**10m**	4	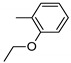	31	97	143–145
15	**10n**	4	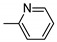	70	98	175–178
16	**10o**	4	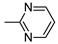	45	90	146–148
17	**10p**	4	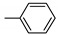	55	90	133–137
18	**10r**	4	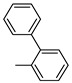	61	95	oil

* Percent purity of the purified product was calculated on the peak area integration during HPLC analysis.

**Table 6:** Activity in Ki ± SD (nM) in vitro tests of trazodone derivatives **10a**–**10k**.

Entry	No.	D_2_	5-HT_1A_	5-HT_2A_	5-HT_6_	5-HT_7_
1	**trazodone**	3650 ^*^	78 ^*^	16^*^	>10,000 ^**^	1782 ^**^
2	**10a**	116 ± 19	285 ± 42	181 ± 22	1430 ± 294	157 ± 13
3	**10b**	1652 ± 203	1547 ± 314	969 ± 114	36,380 ± 5817	7415 ± 625
4	**10c**	122 ± 8	459 ± 36	178 ± 25	3249 ± 401	174 ± 34
5	**10d**	152 ± 17	593 ± 81	6713 ± 952	9459 ± 1138	539 ± 68
6	**10e**	137 ± 11	16 ± 2	342 ± 48	1945 ± 281	278 ± 19
7	**10f**	522 ± 71	49 ± 4	2600 ± 318	3573 ± 563	2595 ± 359
8	**10g**	362 ± 17	27 ± 3	234 ± 46	2062 ± 173	435 ± 61
9	**10h**	310 ± 42	19 ± 3	77 ± 14	576 ± 82	196 ± 29
10	**10i**	195 ± 11	7 ± 2	417 ± 52	4736 ± 687	993 ± 214
11	**10j**	57 ± 6	4 ± 1	841 ± 105	11,530 ± 2114	219 ± 36
12	**10k**	21 ± 3	9 ± 2	343 ± 21	3497 ± 537	1024 ± 184
13	**10l**	350 ± 54	826 ± 91	3903 ± 432	5617 ± 743	8297 ± 1351
14	**10m**	1643 ± 219	9 ± 2	1540 ± 225	4816 ± 581	251 ± 52
15	**10n**	10 ± 2	18 ± 3	1718 ± 193	4637 ± 341	1049 ± 91
16	**10o**	202 ± 31	104 ± 12	10,620 ± 1954	7224 ± 827	5569 ± 438
17	**10p**	1526 ± 116	28 ± 4	391 ± 27	3328 ± 197	404 ± 67
18	**10r**	191 ± 14	20 ± 3	328 ± 49	1188 ± 165	19 ± 3

* [23], ** [24].
